# Age Group Differences in Household Accident Risk Perceptions and Intentions to Reduce Hazards

**DOI:** 10.3390/ijerph16122237

**Published:** 2019-06-25

**Authors:** James Morgan, John Reidy, Tahira Probst

**Affiliations:** 1 Psychology Group, Leeds Beckett University, Leeds LS1 3HE, UK; 2 Department of Psychology, Sociology and Politics, Sheffield Hallam University, Sheffield S1 1WB, UK; J.G.Reidy@shu.ac.uk; 3 Department of Psychology, Washington State University, Vancouver, WA 98686, USA; probst@wsu.edu

**Keywords:** household accidents, risk perception, worry, control, optimism, risk attitudes, efficacy appraisals, behavioural intentions

## Abstract

Very little is known about the extent to which seemingly vulnerable younger and older adults appraise household risks and relatedly whether safety information focused on raising risk awareness influences intentions to reduce hazards in the home. The present study assessed age differences in accident experience, risk attitudes, household accident risk perceptions, comparative optimism, personal control, efficacy judgements, and intentions to remove household hazards. It also examined the predictors of these intentions. Thirty-eight younger adults (aged 18 to 25) and forty older adults (aged 65 to 87) completed study booklets containing all measures. There were significant age group differences for all accident experience and risk-related variables. Younger adults experienced more accidents, had riskier attitudes, and had significantly lower cognitive risk perceptions (i.e., they were less likely to be injured due to a household accident). They also had lower affective risk perceptions (i.e., they were less worried) about their accident risk and perceived more personal control over the risk compared with older adults. Young adults were comparatively optimistic about their risk while older adults were pessimistic. Older adults had higher response efficacy and intentions to reduce hazards in the home. Only worry, response efficacy, and risk attitudes predicted intention, however, these relationships were not moderated by age or efficacy appraisal. Although tentative theoretical and practical implications are presented, further research is required in order to better understand the objective and subjective risk associated with household accidents, and to determine the factors that may improve safety, particularly for those most vulnerable.

## 1. Introduction

In the UK, and especially in England, it is difficult to ascertain the objective risk of having an accident in the home. This is because the government-funded data collection mechanism, the Home & Leisure Accident Surveillance System, ceased to operate in 2002. However, based on figures from 2002, the Royal Society for the Prevention of Accidents (RoSPA) estimates that more than 4000 people die every year as a result of household accidents. For those injured at home who attend hospital for treatment, the total cost to the national economy is thought to be considerable, estimated at £45.63 billion per year, £15 billion more than for road traffic accidents (RoSPA).

Although the current objective risk of household accident involvement for English adults is difficult to ascertain, perhaps a proxy indicator is Accident and Emergency (A&E) Department admissions data. Hospital Episode statistics for 2014–2015 [1] show that 0.7% of attendances were the result of an assault, 1.2% after a road traffic accident, 1.9% because of a sports injury, 3.0% ‘unknown’, 19.9% due to an ‘other accident’, and 72.7% recorded as ‘other’. Household accidents are not identified in this typology. However, it is assumed with caution that they would be classified as ‘other accident’ or ‘other’. This means that it is also plausible that household accidents may account for a substantial proportion of A&E attendances. Data from the U.S. where more detail is recorded suggests that 20% of emergency room visits are due to “injury or poisoning” [2], however it is also unclear how many injuries occur in the home. 

Demographic categorization of UK Hospital Episode Statistics [1] show that 20–24 year-olds attend A&E more than any other adult age group. For example, between 2014 and 2016 1.6 million attended annually, representing 8.1% of total attendances. Young adults aged 18 and 19 are also identified as attending A&E often, accounting for 1.4% and 1.5% of total admissions respectively. Although the number of older adults attending A&E seems to be lower, when the numbers are adjusted to account for age group population size, rates of attendance are almost comparable for those over 65 years of age, and exponentially increase with age. The highest adjusted A&E attendance rates for all adults are in the over 80s age group [3]. 

This apparent pattern of age-related risk has been substantiated to some extent by publicised efforts to reduce accident frequency in older adults. There have been a number of recent household risk awareness campaigns specifically aimed at the older age group, (e.g., ‘Stand Up, Stay Up’, Department of Health, 2016–2019), although to-date no initiatives have addressed household accident risk for younger adults. In contrast, campaigns focused on reducing injuries in young adults have tended to target non-household accident risk factors such as driving behaviour, alcohol consumption, etc. (‘Young Drivers Campaign’, ROSPA, ongoing; ‘It’s the Drink Talking’, Alcohol Concern charity, ongoing). The central component of most safety campaigns, including those aimed at reducing household accident frequency, is the provision of accident statistics (e.g., “people aged 65 or over accounted for 7475 deaths as a result of an accident of which 49% were due to a fall”) in an attempt to communicate objective risk, and to increase subjective risk perceptions. Statistics are often provided alongside information concerning common causal factors (e.g., “Physical ability and lack of mobility, environmental hazards, a previous history of falls”), and in some cases, suggested behavioural solutions deemed to reduce the risk (e.g., “removing trip hazards, ensuring areas of the home are adequately lit”).

Research evaluating the success of campaigns aimed at reducing household accidents is lacking. Also, there is a dearth of studies on the role of risk perception in this risk domain, and on the factors most likely to influence people’s willingness to engage in household accident risk-reducing behaviour, such as removing hazards around the home. Research suggests that theory-based campaigns are more effective in promoting health-protective behaviour compared to atheoretical ones [4]. Given that psychological factors are likely to play a role in individuals’ household accident risk reducing behaviour, it is important that behavioural interventions utilise relevant psychological research. 

Therefore, to address this need the present study sought to adopt a domain-specific approach by evaluating levels of, and potential similarities or differences in, cognitive and affective household accident risk appraisal (as well as risk attitudes, personal control, accident experience, and efficacy judgements) in younger and older adults. To assess cognitive risk perception, we purposely utilised a comparative optimism scale to enable us to also consider whether the different age groups had varying levels of optimism (or pessimism) about their risk. Alongside other variables of interest, we also examined the extent to which these risk perceptions were associated with intentions to remove household accident hazards.

We propose eight research questions to be tested based on our review of the wider social and health psychology literature. In formulating these research questions, we seek to address a number of substantial gaps in the current knowledge base concerning the objective and subjective risk of household accidents for seemingly vulnerable younger and older adults, a lack of evaluation studies assessing the effectiveness of risk awareness safety campaigns, and inconsistent findings concerning the role of risk appraisal and other related factors in predicting health protective behaviour. 

Below we begin our review of the literature by first discussing the distinction between cognitive and affective perceptions of risk and how these relate to health protective behaviors. We then describe and delineate the potential influence of domain-specific and general risk attitudes. We next acknowledge the importance of considering the existence and influence of health (cognitive) risk perception bias (unrealistic comparative optimism) and potential boundary conditions (personal control, accident experience, and closeness of referent). Finally, we briefly examine the literature on efficacy appraisals (self-efficacy and response-efficacy) and consider how these variables might influence the relationship between risk appraisals and protective health behaviour, specifically intentions to remove household accident hazards. 

### 1.1. Risk Perception and Health Protective Behaviour: Differentiating Cognitive (Likelihood) and Affective (Worry) Perceptions

It is likely that risk awareness campaigns originate from social and health psychology theories. These include the parallel process model (PPM; [5]), the health belief model (HBM; [6]), protection motivation theory (PMT; [7]), the psychometric paradigm (PP; [8]), the precaution adoption process model (PAPM; [9]), the extended parallel process model (EPPM; [10]), and the prototype/willingness model (PWM; [11]), which assume that risk perception plays an important role in determining behaviour. Although the assumed role of risk perceptions in predicting initiation and maintenance of health behaviors matches conventional wisdom, empirical support has been mixed [12,13,14,15,16,17,18,19,20]. 

According to Ferrer, Portnoy, and Klein [14], the failure to obtain reliable effects may be due in part to issues related to the conceptualization, operationalization, and measurement of risk perceptions. Specifically, these authors, and others (see [21]) argue that there has been a lack of a consistent distinction between cognitive risk perceptions and affective feelings of worry in the measurement of perceived vulnerability to negative health outcomes. Cognitive risk perceptions are individual judgments regarding susceptibility to a particular negative condition or outcome and are typically measured by asking about likelihood or probability, e.g., “How likely are you to be injured at home in the future?” Affective perceptions refer to how anxious or worried an individual is about an outcome using questions such as “How worried are you about being injured at home in the future?” Although it has been suggested that cognitive and affective risk perceptions work in parallel to guide behaviour [22] the majority of studies have focused only on the influence of cognitive risk perceptions [12,16]. A number of authors have argued that they are distinct constructs that are only moderately correlated [23,24,25] and therefore may drive behaviour in different ways [26].

### 1.2. The Potential Influence of Risk Attitudes

Despite the conventional assumption that some people are generally more willing to take risks than others, within the research area there has been a lack of consensus surrounding the existence of a general attitude towards risk (see [27,28,29,30]). Research findings emphasizing the within-person variability of risk attitudes and behaviour across situations has resulted in a domain-specific view of trait risk taking, reflected in the popularity of the Domain-Specific Risk-Taking (DOSPERT) scale [27,30]. The DOSPERT measures risk-taking attitudes by asking respondents to rate their likelihood of engaging in risky activities within six different risk domains: (i) ethical; (ii) gambling; (iii) investment; (iv) health/safety; (v) recreational; and (vi) social (or in a revised version—five domains where gambling and investment are combined into one ‘financial’ domain). 

Contrary to the assumption that stable risk preferences only exist within specified domains of risk, in a recent study, Highhouse et al. [31] applied bifactor analysis to a large sample of participant responses on the revised version of the Domain-Specific Risk Taking [27], and found evidence for the existence of a General Risk Factor (GRF), in addition to the five residual factors represented by the DOSPERT domains. This study also suggests that matching the level of specificity of risk attitude predictors and criterion variables is important in the measurement of trait risk propensity. For the most part domain-specific factors were the strongest predictors of outcomes theoretically contiguous in their nomological network (e.g., the DOSPERT residual was the best predictor of general health measured using Medical Outcomes Study short-form general health survey, [32]) while the GRF was a stronger predictor of the broader multifaceted construct, counter-productivity. As such, the study authors suggest that the GRF may be a superior predictor of other risky real-world behaviors that are not represented by one of the specific DOSPERT facets. It is our assertion that an alternative interpretation of this relationship is that GRF may (negatively) predict engagement in multifaceted health-protective behaviours, including the removal of household hazards (see *Research Question 8*). 

Although psychological research has tended to suggest that older adults are risk avoidant (e.g., [33,34,35,36]), in line with other studies utilising the DOSPERT (e.g., [27,30]), Rolison et al. [37] found that the relationship between age and level of risk attitude may also be specific to risk domain. Their correlational study assessed domain-specific risk taking attitudes across the adult lifespan (18–93 years of age). In line with previous studies of financial risk taking [38,39], they found that risk attitudes in the financial domain reduced more sharply in later life. Social risk attitudes increased from young to middle age, before reducing steeply in older age, whereas recreational risk attitudes decreased markedly from young to middle age but less so in later life. Within both the health and ethical domains risk attitudes were high in younger adults and declined steadily across the lifespan. Although Rolison et al. [37] demonstrate that the correlation between age and risk attitudes may be situationally specific, the extent to which younger and older adults may differ in each domain (and in general) remains unclear. We sought to assess this in the present study:

*Research Question 1 (RQ1)*: Are there age group differences in domain-specific, and general risk attitudes?

### 1.3. Health Risk Perception Bias

Although recent models of risk perception and decision making include an affective or experiential component, classic health behaviour theories define risk perception as deliberately-derived judgments (cognitive appraisals), and as such, measures have required respondents to either provide absolute (e.g., percentage probability of contracting a disease) or comparative (probability compared to others) likelihood estimates. The relationship between deliberative risk perception and health outcomes is complicated by research evidence which suggests a common bias exists towards inaccurately low risk perceptions, a phenomenon termed “unrealistic optimism” [40]. The accuracy of risk perceptions also depends on measurement. For example, a person’s absolute risk perceptions can be accurate (e.g., that they have a high risk of contracting a disease) but they may have an unrealistically optimistic comparative risk perception (e.g., that they have a lower risk than someone of the same age and gender). 

Findings concerning the implications of low risk perception or unrealistic optimism have been mixed [41]. While a number of studies have found that unrealistic optimism can be associated with better health outcomes [42,43,44], in contrast, other studies have revealed a link with negative health outcomes [26,45], and in some studies unrealistically optimistic participants have been shown to engage in less risk mitigating health protective behaviours [46,47].

Although there is a scarcity of research on age differences in household accident risk behaviour, research on the cultural and social influences on how risks are perceived and reacted to (see [48,49]) suggest that the comparative personal risk estimates of older adults may differ from those of younger adults. Although previous findings are mixed, it is also plausible that these age differences may play a role in subsequent protective behaviour with respect to the removal of household hazards. Although no studies have examined age group differences in the risk appraisal of household hazards, Ostberg [50] and later Weyman and Clarke [51] developed a method for assessing differences in comparative subjective risk between work groups in the forestry and mining industries. Workers at differing proximity from operational risk were presented with paired pictorial depictions of typical scenarios containing hazardous behaviour and were asked to make a judgment about which was more ‘dangerous’. A judgment was required for every possible pairing of scenarios and with a reference vignette depicting a non-operational hazard. By holding the reference item constant in the analyses, the authors were able to calculate the rank order of scenario risk perception for each group of workers as well as risk magnitude scores. Ostberg [50] found no significant differences in risk perception between forestry personnel groups at the level of rank order but notable differences in perceived risk magnitudes. Specifically, workers closest to operations (e.g., workmen and supervisors) underestimated risk, while more distal groups (e.g., senior managers and safety officers) overestimated risk. Although Weyman and Clarke [49] also found limited variability in the rank order of risk perceptions between groups of miners, findings concerning patterns of relative perceived risk magnitude were the reverse of Ostberg’s in that they were greater for those closest to operational risks. The authors of both studies explain the differences between personnel groups with reference to experiential influences associated with organizational role and an apparent interaction with cognitive availability effects such as habituation and familiarity with risk. 

Weyman and Clarke [49] emphasized the advantages of using pictorial representations of risk rather than narrative descriptions. They argue that images help contextualize the risk and reduce ambiguity about the nature of the hazard. Despite these advantages, the accompanying method of collecting paired comparative risk comparisons is somewhat time consuming, requiring 28 individual comparisons in the Weyman and Clarke task. Our study involved the recruitment of elderly participants from a care home where there were restrictions on the amount of time available for participation. Therefore, we deemed the use of such a method impractical. Instead we chose to combine the pictorial depiction technique with the *direct* approach of measuring comparative optimism (or pessimism) at the group level. The *direct *approach uses a single scale to ascertain how much higher or lower a person estimates their personal risk of experiencing an event is, compared with that of an average person with the same demographic characteristics. 

While there is a long history of using comparative judgment tasks to assess how optimistic people are when they make cognitive risk perception appraisals, the measurement of affective risk perception is relatively nascent, and therefore research studies have not adopted the same comparative measurement framework. Due to the lack of a methodological precedent we chose to assess age differences in health risk perception (with regards to household accidents) using a comparative measure of cognitive risk appraisal (also allowing us the possibility to examine the presence of optimistic bias), and a non-comparative measure of affective risk appraisal:

*Research Question 2 (RQ2)*: Are there age group differences in comparative risk appraisals (cognitive risk perception), and affective risk perception across the eight HRI household hazard images? 

*Research Question 3 (RQ3)*: Are there age group differences in comparative optimism when making cognitive risk judgements? 

In a major review of the literature, Helweg­Larsen and Shepperd [51] highlighted several boundary conditions for unrealistic comparative optimism. People show less unrealistic comparative optimism at the group level when (a) events are not under personal control, (b) people have experienced the event previously, (c) the referent for the comparison judgment is a ‘close other’ as opposed to ‘distant other’, and (d) they receive proximal feedback. We sought to further investigate potential age group differences in personal control, household accident experience, and closeness of a referent, and their relationship with (unrealistic) optimism (comparative cognitive risk perception).

*Research Question 4 (RQ4)*: Are there age group differences in the boundary conditions for comparative optimism; personal control (in relation to the HRI images) and accident experience, and is there a main effect of closeness of referent on optimism or an interactive effect with age?

*Research Question 5 (RQ5)*: Do the boundary conditions personal control and accident experience significantly predict optimism, and if so are these relationships moderated by age? 

### 1.4. The Moderating Role of Efficacy Appraisals

According to the EPPM [10] the impact of risk appraisals on behavior is moderated by *efficacy appraisals*. Efficacy appraisals represent a person’s evaluation of their ability to manage a specific hazard and is usually operationalised using measures of self-efficacy (confidence in their ability to enact the recommended behavior) and response efficacy (the perceived capacity for the recommended behavior to protect them from the hazard).

The EPPM suggests that when risk appraisal is high or is heightened but efficacy appraisals remain low, people are more likely to employ emotion-focused coping which involves attempting to reduce the immediate emotional response to the hazard [10,52]. However, when risk appraisal and efficacy appraisals are high people adopt problem-focused coping where they not only recognise the hazard exists, but also that they can take steps to alleviate it.

Although these theoretical propositions are clear, they have lacked empirical support [21,53]. However, a recent meta-analysis of experimental evidence conducted by Sheeran and colleagues [21] concluded that overall studies show heightened risk appraisals have small but significant effects on both intentions and behaviour and these effects were increased when efficacy appraisals were also enhanced. 

*Research Question 6 (RQ6)*: Are there age group differences in efficacy appraisals (self-efficacy and response-efficacy) and intention to remove household accident hazards (after the presentation of risk awareness campaign information)?

*Research Question 7 (RQ7)*: Do cognitive or affective risk perceptions predict intention to remove hazards in either age group or in the study sample as a whole, and are these relationships moderated by efficacy appraisals? 

### 1.5. Predicting Intentions to Remove Household Accident Hazards

Subsequent to our review of relevant literature and in addition to research questions 1–7, our final goal was to identify the predictors of intention to remove hazards for each age group and our sample as a whole:

*Research Question 8 (RQ8)*: Do the additional study variables (i.e., general risk attitudes, personal control, and prior accident experience) significantly predict intention to remove household hazards, and are these relationships moderated by age? 

## 2. Method

### 2.1. Participants

The present study included 78 volunteers; 38 younger adults aged 18–25 years (*M* = 21.08 years, *SD* = 1.99, 20 female), and 40 older adults aged 65–87 years (M = 73.90 years, SD = 6.79, 26 female). Participants were recruited for a study on ‘attitudes towards household hazards’ from a university campus and from a retirement village, both located in the North of England. The support services (packages) offered by the retirement village to residents range from organizing social activities to home cleaning and maintenance, to personal and medical care. Some residents live completely independently while others receive full support. In the case of the older participants in the current study it was ensured that none were receiving a ‘care package’, meaning that they were free from physical or mental illness or impairment.

### 2.2. Design 

The study adopted a quasi-experimental design, comparing participant responses across two specific age groups; young adults, aged between 18 and 25 years, and older adults, aged 65 years or older. The main household accident risk appraisal variables were contained in a Household Risk Inventory developed for the purposes of the study, namely measures of comparative cognitive risk perception, affective risk perception (accident worry), and personal control. Domain-specific and general risk taking attitudes, and accident experience were also measured. Participants were then asked to read an extract from a RoSPA household safety publication, which was followed by measures of self-efficacy, response-efficacy and behavioural intentions to reduce hazards in the home.

### 2.3. Procedure

Participants each completed a study booklet containing the measures on their own in a designated quiet room, either on the university campus or at the retirement village. 

The first page of the study booklet provided participants with information about the study requirements, and an explanation of their ethical rights including a request for their consent. Demographic questions and measures of risk-taking attitudes and accident experience followed. Dependent on age group, the next pages of the study booklet included one of two versions of the Household Risk Inventory (HRI, see below). The two versions of the HRI differed on only one aspect; the measure of ‘closeness of a referent’, with the younger adults receiving one version and the older adults the other (see below for further explanation). After the HRI participants were presented with a brief extract from a Royal Society for the Prevention of Accidents information document about accidents in the home and how to reduce them. Participants were requested to read the information and then respond to three items about their self-efficacy, response efficacy, and intentions to remove hazards in their home. On the final page of the booklet, participants were thanked and debriefed. All data collection procedures were approved by the Institutional Review Board at Sheffield Hallam University (IRB protocol# Hallam/Psych/12-13/Manning).

### 2.4. Measures

#### 2.4.1. Risk Taking Attitudes 

Attitudes towards risk taking were measured using an abridged, and adapted, 24-item version of the original Domain-specific Risk-Attitude Scale [30]. The measure requires respondents to indicate the likelihood that they would engage in an activity or behaviour described in 40 statements covering six content domains: Investment (I), gambling (G), health/safety (H), recreational (R), ethical (E), and social decisions (S). The measure uses 5-point response scales ranging from *very unlikely *(1) to *very likely* (5). High scores represent an increased likelihood of engaging in risk taking behaviours or activities. For the purposes of the present study, the subscales relating to investment, gambling, and ethical domains were omitted. In addition, a number of the statements were reworded to ensure that they were appropriate for the two study populations (i.e., young and old UK residents). For example, a statement including a reference to ‘a tornado’ was changed to ‘extreme weather’. Another statement referring to a parent, ‘father’ was altered to read ‘a close family member’. Sub-scale reliabilities were acceptable (H, *α* = 0.79; R, *α* = 0.88; S, *α* = 0.71). Following Highhouse et al. [31], who identified a general risk attitude factor, we also created a composite measure of risk attitude by aggregating domain-specific item responses (*α* = 0.91). 

#### 2.4.2. Accident and Injury Experience

A measure of recent accident experience followed. The format was based on a questionnaire used by Warrington and Wright [54] to gather child accident frequency and typology data from parents. The present study required participants to reveal how many times they had experienced three types of common household accidents (burns/scalds, a bad fall, DIY-related) and any other accident over the last 12 months. The responses were aggregated to form one 12-month accident frequency variable. 

Participants were also asked to report the number of times they had suffered major injuries in the previous 4 years. The injuries presented were; a broken bone (leg/foot, arm/hand, skull, or other), being knocked unconscious, cut, or burnt. Frequency data for these major injuries were combined to form one variable representing the number of major injuries experienced in the last 4 years.

#### 2.4.3. Household Risk Inventory (HRI)

The method used by Ostberg [50] and Weyman and Clake [49] was adapted for use in the present study. Participants were presented with eight images depicting household hazards alongside a caption describing the content of each picture. The choice of pictorial images was based on common hazards identified by RoSPA. The captions included the following descriptions of each hazard; “objects left on a stairway”, “a rug positioned in two doorways”, “hammering a nail into the wall”, “a drink spilled on a smooth floor”, “carrying three mugs of tea”, “electricals near the bathroom sink”, “standing on a step ladder to reach a high point”, “pan handles pointing outwards”. Google image searches were utilised to find appropriate non-copyrighted pictures. Accompanying each image were five items. Cognitive risk perception was measured using a method previously utilised to operationalise comparative optimism. The following item required participants to judge their level of cognitive risk compared with a person with similar characteristics: “How probable do you feel it is that you would experience an accident in the situation above, compared to another person of your age?” on a 5-point response scale ranging from 1 (*not at all probable*) to 5 (*very probable*). Affective risk perception was measured using the following item: “How worried do you feel when thinking about the probability of an accident occurring to you in this situation?” on a 5-point response scale ranging from 1 (*not at all worried*) to 5 (*very worried*). Personal control (a potential boundary condition for unrealistic optimism) was measured using the item: “How much control do you feel you have over an accident occurring to you in the situation portrayed in the picture?” on a 5-point response scale ranging from 1 (*complete control*) to 5 (*no control*). 

In addition to accident experience and personal control, ‘closeness of a referent’ was identified as a potential boundary condition for unrealistic optimism. As such a further item was also included after each HRI image. Participants were asked to provide an indication of their comparative optimism with respect to members of the alternative age group as the reference point. For example, in the older adult group, participants were asked: “How probable do you feel it is that you would experience an accident in the situation above, compared to an adult aged 18–25?” on a 5-point response scale ranging from 1 (*not at all probable*) to 5 (*very probable*). 

#### 2.4.4. Efficacy and Intentions to Remove Risk

All participants were asked to read the same general 450-word passage about accidents in the home. It included information on A&E admission statistics (from Hospital Episode Statistics), the most common types of accidents, suggested prevention strategies, what people should do if they have an accident in the home (from the Royal Society for the Prevention of Accidents website). Following the presentation of this information, we gathered measures of efficacy and behavioural intentions adapted from Epton and Harris [55]. 

Self-efficacy was measured using a single item; “for me, removing the number of risks around my home would be…” and a 4-point response scale ranging from 1 (*very easy*), 2 (*quite easy*), 3 (*quite difficult*), to 4 (*very difficult*). Responses were reverse scored so high scores represent more self-efficacy. Response efficacy was also measured using one item: “How confident are you that removing potential risks around the home will actually prevent accidents” with a 5-point response scale ranging from 1 (*not at all*) to 5 (*completely confident*). Behavioural intention was measured using the item “I intend to remove the number of potential risks around my home in the next week” and a 4-point scale ranging from 1 (*definitely don’t*), 2 (*probable don’t*), 3 (*probably do*), to 4 (*definitely do*).

## 3. Results

The means and standard deviations for all study variables are presented in Table 1. To examine whether there were group differences in domain-specific or general risk attitudes (RQ1), a between-participants multivariate analysis of variance (MANOVA) with age group as the independent variable (IV) and domain-specific risk attitudes as the dependent variables (DV) was conducted. There were significant differences between age groups for all three risk attitude domains (health/safety, recreational, and social decisions). Older adults had less risky domain-specific attitudes (multivariate ANOVA, Wilks’ λ = 0.35, *F*(3.70) = 43.80, *p* < 0.001, η_p_^2^ = 0.65; for univariate ANOVAs all *F*s > 35.04, all *p*s < 0.001, η_p_^2^s ≥ 0.33). There was also a significant difference between the groups on the total score for attitudes indicating that older adults had lower general risk attitudes (*t*(75) = 9.27, *p* < 0.001, *d* = 2.11).

The differences between younger and older adults in cognitive risk perceptions, and affective risk perceptions for each of the HRI images (RQ2) were analysed through two separate one factor MANOVAs. In each of these, age group was entered as the between-participants factor and the cognitive or affective risk ratings for each of the 8 risk scenarios as the dependent variables.

The first of these, for cognitive risk perception, revealed a significant multivariate difference between the groups (Wilks’ λ = 0.78, *F*(8.69) = 2.51, *p* = 0.019, η_p_^2^ = 0.23). Inspection of the univariate ANOVAs indicated that there were significant between group differences for rug, nail, spillage, tea and reaching on a ladder hazards (all *F*s(1.76) > 5.35, all *p*s < 0.024, η_p_^2^s ≥ 0.066) which the older group rated in all cases as more risky than the younger group (see Table 1). 

Similarly, for the affective risk ratings there was a significant multivariate difference between the two age groups (Wilks’ λ = 0.52, *F*(8.69) = 7.89, *p* < 0.001, η_p_^2^ = 0.48). The univariate ANOVAs suggested that in this case there were significant differences between the two groups for all hazards (all significant* F*s > 9.47, all *p*s < 0.003; η_p_^2^s ≥ 0.11) except nail (*F*(1.76) = 3.71, *p* = 0.058, η_p_^2^ = 0.05) and electricity (*F*(1.76) = 0.63, *p* = 0.429, η_p_^2^ = 0.01). With the exception of these two hazards, older adults were significantly more worried than younger adults about the risks depicted in the other six images. 

Given that there were differences between the two groups in terms of cognitive risk appraisals, analyses were conducted to establish whether there were any group differences in optimism/pessimism towards risk (RQ3). To this end, and to address the remaining research questions, the risk perception ratings were aggregated across the eight household images. For these aggregated ratings a score below 3 represents an optimism bias (i.e. feeling less at risk compared with ‘an other’ from the same group) and a score above 3 represents a pessimism bias (feeling more at risk). One sample t-tests showed that the younger group had an optimism bias for cognitive risk estimates (mean of 2.77, *t*(37) = 2.74, *p* = 0.009, *d* = 0.44) whereas the older group had a pessimism bias for cognitive risk estimates (mean of 3.26, *t*(37) = 2.22, *p* = 0.032, *d* = 0.36). Additionally, the younger group were significantly more optimistic than the older group (*t*(76) = 3.38, *p* = 0.001, *d* = 0.77).

To examine group differences in the boundary conditions for unrealistic optimism (*RQ4*) a MANOVA was conducted with accidents in the last year, and last 4 years, and mean personal control (aggregated across all HRI images) as the dependent variables. This revealed a significant multivariate difference between the groups (Wilks’ λ = 0.70, *F*(3.74) = 10.81, *p* < 0.001, η_p_^2^ = 0.31). Examination of univariate ANOVAs showed that whilst the younger group had significantly more accidents in the past 12 months and past 4 years, they also rated themselves as having greater personal control over HRI risks (all *F*s > 6.05, all *p*s ≤ .016, η_p_^2^s ≥ 0.07) see Table 1 for means and SDs).

**Figure 1:**
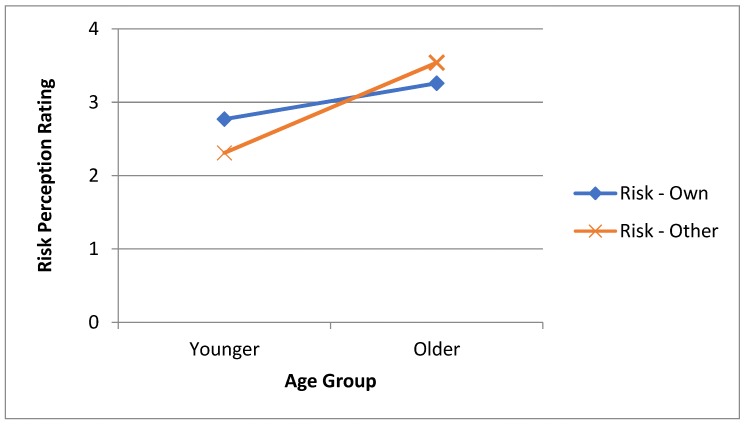
Means ratings for overall own risk and other risk ratings for the younger and older age groups.

To establish whether closeness of referent group and age have an impact on risk appraisals (*RQ4*) an ANOVA with one between-participants factor of age group and one within-participants factor of reference group (own vs other) was conducted. This revealed no significant main effect of reference group (*F*(1.76) = 2.13, *p* = 0.15, η_p_^2^ = 0.03) but there was a significant main effect of age group (*F*(1.76) = 45.47, *p* < 0.001, η_p_^2^ = 0.37) and importantly a significant interaction (*F*(1.76) = 38.05, *p* < 0.001 η_p_^2^ = 0.33). To explore this significant interaction (see Figure 1) paired-samples *t*-tests were conducted separately for each age group and these showed that for the younger age group ratings of risk compared to the other group (mean of 2.31) were significantly lower than ratings compared with own group (mean of 2.77; *t*(37) = 5.63, *p* < 0.001, *d* = 0.91) whereas there was the opposite pattern for the older age group with risk ratings for comparisons with own group (mean of 3.26) being lower than those comparing to the other group (mean of 3.54; *t*(39) = 3.22, *p* = 0.003, *d* = 0.50). 

*RQ5* suggests that the boundary conditions of perceived personal control over risks and accident experience may be predictive of cognitive perceptions of risk. To examine this, moderation analyses were conducted to assess whether any such relationships exist, and whether these relationships are moderated by age group. A hierarchical regression analysis was conducted with age, accidents in the past 12 months, and four years, and mean HRI perceived control entered in step 1 and the product terms of the three predictors with age group entered in the analysis in step 2. The dependent variable was cognitive risk perception (see Table 2 for detailed results for three hierarchical regressions). 

This analysis showed a significant model in step 1, with perceived control and age group as significant predictors of cognitive risk perception. Both of the accident predictors were non-significant. The introduction of the interaction terms in step 2 of the analysis yielded no significant increase in R^2^ indicating that there were no moderated relationships by age group.

The literature suggests that cognitive risk perceptions may be moderated by efficacy appraisals in predicting behavioural intentions (RQ7, e.g., see [21]). To examine this possibility as well as the possibility of a similar moderated relationship involving affective risk appraisals a hierarchical regression was conducted with cognitive and (HRI aggregated) affective risk perceptions, self-efficacy and response efficacy entered in step 1 and the product of the efficacy appraisals with cognitive and affective risk perception entered as interaction terms in step 2, the dependent variable being intentions to remove hazards. This analysis suggested that there was a significant effect of affective risk perception and near significant effect of response efficacy in Step 1 but no significant effects of self-efficacy and cognitive risk perception and no significant interactions, i.e. no significant increase in R^2^ at step 2 of the analysis. 

It should be noted with reference to *RQ6* that there was a significant multivariate difference between the age groups in efficacy appraisals and intentions to remove hazards (Wilks’ λ = 0.73, *F*(3.73) = 8.93, *p* < 0.001, η_p_^2^ = 0.27) with significant univariate differences in response efficacy (*F*(1.75) = 14.40, *p* < 0.001, η_p_^2^ = 0.16) and intentions (*F*(1.75) = 17.238, *p* < 0.001, η_p_^2^ = 0.19) but not self-efficacy (*F*(1.75) = 0.78, *p* = 0.38, η_p_^2^ = 0.01). The younger age group had lower response efficacy ratings and also lower intentions to remove hazards. Separate hierarchical regression analyses for the different age groups showed no significant main effects or interactive effects involving efficacy appraisals and cognitive or affective risk perceptions in predicting intentions (all *F*s < 2.66, all *p*s > 0.083).

The analyses to address *RQ7* is suggestive of response-efficacy and worry being predictors of intentions to remove hazards. A final analysis addressing *RQ8* examined whether similarly general risk attitudes (aggregated from the domain-specific attitude scores), perceived control over hazards, and accident experience were predictors of intentions and whether they were moderated by age. As before, the product terms of these predictor variables with age were entered into step 2 of a hierarchical regression analysis. As with the previous analyses there was a significant equation in step 1 of the analysis indicating only a significant relationship between general attitudes to risk and intentions to remove hazards and all other predictors were non-significant. There were no significant interactive effects at step 2.

## 4. Discussion

The present study attempted to address various theoretical and practical knowledge gaps by exploring similarities and differences in subjective risk perceptions and related risk attitudes and beliefs in two seemingly vulnerable groups (young adults and older adults). The current research also explored the relationships between these variables and tested whether they predict intentions to engage in domain-specific health protective behaviour (the removal of hazards in the home).

### 4.1. Answers to Our Research Questions

*RQ1.* Our findings suggest numerous significant age group differences. Our first research question (*RQ1*) concerned the possible effect of age on domain-specific and general risk attitudes, factors thought to influence risky behaviours, contingent on the level of congruence in specificity of the attitudinal measure and the targeted behaviour [31]. In line with previous research suggesting older adults are more likely to be risk avoidant (see [33,34,35,36]), older adults (aged 65 and over) in our study exhibited lower levels of general risk attitudes compared with the younger adult group. 

*RQ2.* Our results suggest that there were also significant differences in the subjective risk estimates of older and younger adults with regards to the specific household accident hazards depicted in the Household Risk Inventory (HRI). Older adults perceived significantly more cognitive risk for rug, nail, spillage, tea, and reaching on a ladder hazards, and worried significantly more about all depicted hazards except nail, and electricity (*RQ2*). This was in contrast to younger adults, who perceived low levels of both cognitive and affective risk. 

*RQ3.* Measuring cognitive risk perception using a *direct* form of a comparative optimism scale (at the group level) allowed us to determine whether cognitive risk judgments were optimistic (*RQ3*). Our findings suggest that young adults’ low risk perception ratings represent comparative optimism about their likelihood of being injured in various hazardous household scenarios (compared with the likelihood of ‘an other’ person of the same age). Although it is not common for researchers to interpret responses to comparative optimism scales in relation to levels of pessimism, our findings also suggest that the older adults’ high levels of risk perception for the majority of hazards (see above) were comparatively pessimistic. 

In contrast to our findings, previous research by Vanderzanden and Ruthig [56] found that comparative optimism seems to be a robust tendency in older adults. While we found that older adults were comparatively pessimistic about their household accident risk, Vanderzanden and Ruthig found that for estimates of future health (i.e., over the next year) very few older adults in their study expected to have worse health (as opposed to better health or no change in health—representing optimism or realism respectively). Similarly, of particular relevance to the present study is the research on the risk appraisals of older people with respect to future injuries. Ruthig et al. [57] found community-living older adults estimated that they were comparatively less likely to suffer a hip fracture and Dollard et al. [58] showed that older individuals were comparatively optimistic about their chances of having a fall in the following year. 

Our findings regarding age group differences in comparative optimism seem surprising in light of previous results suggesting it is a pervasive bias, universal across age groups. Despite this, review papers on optimistic bias (see [51,59,60]) imply that comparative optimism may be context- or relevance-specific. Therefore, our inclusion of measures of possible boundary conditions for unrealistic optimism allow us to determine whether these moderators may explain our age group differences. For example, greater perceived control has been found to predict lower personal risk estimates [51,56]. Previous experience of the target outcome and the closeness of the comparison referent, in terms of how similar they are to the respondent, have also been shown to reduce comparative optimism [51].

*RQ4* and *RQ5.* The preliminary analysis of boundary conditions (*RQ4*) suggested that significant age group differences in perceived personal control may go some way to explain the pattern of comparative optimism responses of younger and older adults, i.e. younger adults were more optimistic and also perceived more control. However, our regression analysis (to test *RQ5*) showed that while personal control and age significantly predict comparative optimism separately, there was no moderating effect of age. Our results regarding the role of previous accident experience also failed to support Helweg-Larsen and Shepperd’s [51] review findings in that the young adults in our study were comparatively optimistic about household accident injuries despite the fact that they had experienced significantly more accidents than older adults. However, ours is not the first study to observe this pattern of results. Dollard et al. [58] found that knowing someone who had experienced a dangerous fall, or even personally suffering a very recent fall, did not prevent older adults’ optimistic estimates about their own risk of falling. 

With regards to the effects of closeness of referent comparison group and age (also *RQ4*), the main effect of age indicates that irrespective of comparison referent, the younger adult group were more optimistic about their likelihood of having an accident compared to the older adults. The interaction effect suggests that this optimism in young adults is reduced when the comparison referent is closer (from their own age group) than when the comparison referent is more distant (from the other, older age group). It appears that this pattern is reversed for older adults who are less optimistic about their risk when a young adult is the referent. In other words, both younger and older adults seem to perceive a greater risk of injury occurrence for older adults. 

*RQ6.* In addition to age group differences in risk appraisal, the current study also sought to assess whether typical household injury campaign information influenced younger and older adults’ efficacy appraisals and behavioural intentions to take remedial action to remove household hazards. Due to equivocal findings in general and a paucity of research specifically in this behavioural domain, we were also interested in the interrelationships between risk appraisals, efficacy appraisals, and intentions. Following the pattern of our previous results concerning age differences in risk attitudes and risk appraisals (comparative optimism and worry), older adults were shown to have significantly higher response efficacy but there was no significant group difference for self-efficacy (*RQ6*). With regards to intentions to remove hazards, the older group had greater intentions than the younger group (*RQ6*). 

*RQ7.* In the regression analysis of older and younger adults’ combined data, response efficacy and affective, but not cognitive, risk appraisals predicted intentions, with higher response efficacy and worry linked to greater intentions to remove hazards (*RQ7*), however the absence of interactive effects suggests that the risk perception-intention relationship was not moderated by efficacy appraisals (*RQ7*). Analyses conducted on each age group separately revealed no significant main effects or interactive effects involving efficacy appraisals and cognitive or affective risk perceptions in predicting intentions (*RQ7*). 

The findings addressing *RQ6* and *RQ7* are somewhat equivocal in the light of previous research in this area. On one hand our findings support the proposition that the role of cognitive and affective risk appraisals should be assessed separately [14] as we found that across both age groups worry about hazards rather than likelihood judgements influenced behavioural intentions. On the other hand, however, there were no moderation effects of efficacy appraisals, which is contrary to the theoretical propositions of the EPPM [10]. Also, although older people rated their likelihood of experiencing an accident as higher and were more worried about hazards than the younger group, and had more response efficacy, and greater intentions, it is somewhat surprising that separate analysis of their responses did not indicate any direct or indirect causal relationships. 

*RQ 8.* Our final research question concerned whether general risk attitudes, personal control, and accident experience are predictive of intention to remove hazards with or without age (*RQ8*). Again, although there were significant age group differences, only general risk attitudes significantly predicted intention to remove household hazards and there were no moderation effects of age. 

### 4.2. Limitations and Future Research Directions

Our study produced a number of surprising results in comparison with previous research findings. Some of these may be due to study design differences or limitations of the present study. For example, it seems that the boundary conditions of perceived personal control or previous experience cannot explain age differences in comparative optimism in the current study. It is likely that this is either due to the way we operationalized these variables and/or because other factors not measured in the current study influenced the household accident risk appraisals of participants. There are additional event characteristics that have been shown to influence optimistic bias, such as (for appraisals of future negative life events) lower event frequency, severity, and the easier it is to imagine a stereotypical victim of the negative outcome [61]. It is likely that the age group differences in risk appraisals in the present study are due to variations in perceived event characteristics between younger and older adults. In particular, although we did not directly measure or manipulate event severity, it is possible that older adults perceived the hazards depicted in the HRI as more severe than young adults did. In line with the findings of Taylor and Shepperd [62] and later Harris et al. [61] it is posited that that older adults felt more threatened by the hazards depicted because physical injuries generally impose more dramatic life changes for the elderly due to declining physiological strength. For example, in keeping with research on human development and ageing, our study findings may reflect the reality that older adults are more likely to experience severe physical outcomes as a result of an accident in the home (e.g., bone breakage due to decreased bone density) and longer recovery times, compared to younger people. This may also explain why younger adults, who had had more recent experience of accidents, were still optimistic about their risk, i.e., they perceived low levels of event severity (and life impact) due to their superior physical condition (e.g., ability to recover). 

Our findings were also at odds with similar studies that found older participants were optimistic rather than pessimistic about their risk. However, plausible reasons for this are that the target events in these studies were not household accident hazards, that the situational context was different for our participants, and also that our method of risk appraisal measurement was distinct, using hazard images as stimuli. In the study conducted by Dollard et al. [58] participants were comparatively optimistic about their risk of falling in the future, and Vanderzanden and Ruthig [56] observed comparative optimism about future health status. The differences in event characteristics alone may account for the contrasting findings, however it is also important to note that the situational context surrounding older adults recruited in these previous studies differed from ours in a number of aspects. Both of the aforementioned studies were conducted outside of the UK (one in Australia and the second in the U.S.) and included only people living independently in the community (i.e., “community-dwelling”). In contrast, our older adults were recruited from a UK retirement village where they live alongside other older adults and are provided with minor support for routine activities. It is possible that event characteristics particular to household accidents, the way we measured comparative optimism, the particular situational context for older adults, the broader UK-specific culture, or an interaction between these factors, may have contributed to the comparative pessimism of older adults in our study and not others. It is also possible that these factors may have influenced older adults’ risk appraisals directly or indirectly by increasing perceived severity. 

An additional surprising finding in the current study was that risk perceptions did not predict intentions to remove hazards, and there were no interactive effects involving efficacy appraisals. Inconsistent findings are not uncommon especially in correlational studies [21]. A recent meta-analysis of experimental evidence conducted by Sheeran and colleagues [21] concluded that heightened risk appraisals have small but significant effects on both intentions and behaviour and these effects were increased when efficacy appraisals were also enhanced. Of particular relevance, however, is that the largest effects were observed when sub-components of risk appraisal (risk perception, anticipatory emotion, anticipated emotion, and perceived severity), response efficacy, and self-efficacy were simultaneously heightened. Although the majority of these components were included in the present study (risk perception/cognitive risk appraisals, anticipatory emotion/affective risk appraisals, response efficacy, and self-efficacy), perceived severity and anticipated emotion were not. We have already suggested that perceived severity may explain age differences in risk appraisals in the present study. It is also possible that perceived severity is an important factor in determining behavioural intentions in this domain, particularly for older adults who, we argue, are likely to regard household hazards as more serious. Two key studies on event characteristics found that the effect of severity on cognitive risk appraisals is driven by heightened vigilance associated with negative affect [61,62]. While the present study findings imply that negative affect may play an important role in the appraisal of risk and influence intentions to reduce household hazards, further research is required in order to better understand how worry operates alongside other important factors, some of which were not included here. Also, because our study was the first to examine the possibility of age differences in this domain, there remains some uncertainty about whether the factors influencing risk perception and behaviour are different for older and younger people. For example, although there were consistent age differences in mean scores for almost all hazard appraisal variables, efficacy judgements, and behavioural intentions, age did not moderate the relationships between these variables.

In addition to the above, it is important to acknowledge other limitations of the current study which should be considered when interpreting the generalizability of the findings and/or planning similar future studies. Firstly, the modest sample size in the present study means that there may have been insufficient power in the analyses to detect all effects and that the significant findings may not be generalizable beyond the small samples. Also, due to procedural restrictions, particularly regarding the time available to access older adults, the current study relied entirely on self-report measures and therefore participant responses may have been subject to self-report bias. 

### 4.3. Practical Implications

As we suggest above, further research is required in this domain in order to determine the causal links between risk perception components, efficacy judgements and protective behaviour, as well as interaction effects involving (perceived) event characteristics, individual differences, and other demographic factors such as age and event exposure.

From a practical perspective, our analysis of participant-reported previous accident experience data suggests that the objective risk of household accidents is higher for younger adults than it is for older adults. We must be cautious in generalizing beyond the populations sampled; however, taken together with accident and emergency attendance statistics, we can assume that household accident risk for younger people is sufficient for us to suggest that future injury reduction campaigns should consider targeting this age group. To date, campaigns have primarily focused on older adults, although the rationale for this is not clear. It is possibly because the UK data that is available indicates elderly people have more accidents. The impact of age-related physical deterioration could also be a influencing factor because, irrespective of actual injury frequency probability, compared with other age groups, the consequences of injury may be considered more severe. In any case, previous household accident reduction campaigns have largely focused on raising awareness of hazards in the home and have also included suggestions on how people can reduce these risks. 

The results of the present study suggest that such campaigns may have achieved some success in that older participants rated the cognitive risk of household hazards as high compared to younger adults, and also had stronger intentions to remove hazards at home. However, our study also showed that irrespective of age, high cognitive risk perceptions did not predict intentions but affective risk perceptions (worry) did. The implication is that accident and injury reduction initiatives aimed at younger or older adults should consider broadening their focus to include content aimed at increasing affective appraisals concerning the consequences of injury. We suggest that it is likely that a combination of factors, some of which were not included in the present study, predict both risk appraisals and protective behaviour in this setting. 

Because this was the first study to explore household accident risk, the practical implications are limited. Policy makers and campaigners seeking to promote health behaviours should ensure they are guided by research, ideally conducted in the specific domain of interest. We have provided some preliminary data which may be useful as a foundation for behaviour change intervention development in this area, however further research is required to complete the first stage of this process – determining what predicts the behaviour (see [63]). 

## 5. Conclusions

This was the first study to explore cognitive and affective household accident risk appraisal. We were specifically interested in whether these appraisals and levels of other related constructs were similar for two seemingly ‘at risk’ age groups; young adults and old adults living in England. Because, to-date, household injury reduction initiatives have been aimed at raising risk awareness in the elderly, we were also interested to see if heightened risk appraisals predicted intentions to adopt health protective behaviours in the form of removing hazards in the home. The findings suggest that younger and older adults do differ in their risk-related attitudes, beliefs and judgments, in that the older group had less risky attitudes, perceived more cognitive and affective risk, and less personal control over household risks. Older adults also had higher response efficacy and intention to remove hazards. Despite this consistent pattern of age group differences, only affective risk appraisals (worry), response efficacy, and in a separate regression analysis, general risk attitudes, predicted behavioural intentions, and importantly these relationships were not moderated by age. Further work is required in order to determine the predictors of protective behaviour in this setting, to evaluate existing campaigns, and guide the development of future interventions for those considered most at risk. 

## Figures and Tables

**Table 1:** Means and standard deviations for younger and older groups for risk perception and worry for each HRI hazard along with risk attitudes, ratings for control, past accidents, overall risk perceptions, efficacy appraisals, and behavioural intentions to remove hazards.

Variable Type	Specific Measure	Young		Old		Effect Size
		*Mean*	*SD*	*Mean*	*SD*	*d*
Risk Attitudes	Risk attitude-recreational	2.68	0.80	1.43	0.61	1.76 ***
	Risk attitude-health & safety	2.72	0.50	1.52	0.49	2.42 ***
	Risk attitude-social	3.31	0.41	2.53	0.68	1.39 ***
	General attitude to risk	8.49	1.52	5.44	1.37	2.11 ***
HRI Risk Domains	Cognitive risk perception:					
	Stairs	2.83	0.95	3.23	1.39	0.34
	Rug	2.63	1.11	3.26	0.91	0.62 **
	Nail	2.71	0.89	3.26	1.16	0.53 *
	Spill	2.80	0.87	3.46	1.10	0.67 **
	Tea	3.06	0.97	3.56	1.21	0.46 *
	Electricity	2.71	0.89	2.74	1.31	0.03
	Reaching on step ladder	2.86	0.85	3.38	1.25	0.49 *
	Pan handles	2.69	0.76	3.05	1.28	0.34
HRI Risk Domains	Affective risk perception:					
	Stairs	2.23	0.97	3.41	1.35	1.00 ***
	Rug	1.86	0.85	3.36	1.14	1.49 ***
	Nail	2.57	1.14	3.05	1.28	0.40
	Spill	2.40	1.09	3.67	1.18	1.12 ***
	Tea	2.80	0.87	3.62	1.11	0.82 ***
	Electricity	3.17	1.04	3.26	1.37	0.07
	Reaching on a step ladder	2.74	1.12	3.69	1.22	0.81 ***
	Pan handles	2.71	1.02	3.51	1.50	0.62 **
Control	HRI Personal control	3.94	0.42	3.59	0.75	0.58 *
Accident Experience	Accidents in last 12 months	2.80	2.46	0.54	0.91	1.22 ***
	Major injuries in last 4 years	0.63	0.91	0.13	0.41	0.71 **
Risk Perception	Risk compared with own age (comparative optimism)	2.79	0.53	3.24	0.73	0.71 **
	Risk compared with other age	2.33	0.48	3.53	0.7	2.00 ***
	Closeness of referent weighting (other age–own age)	−0.45	0.51	0.28	0.57	1.35 ***
Efficacy Appraisals & Intentions	Self-efficacy	3.00	0.69	3.13	0.56	0.20
	Response-efficacy	3.50	0.89	4.21	0.73	0.87 ***
	Behavioural Intentions	2.42	0.76	3.08	0.62	0.95 ***

Groups differ significantly at * *p* < 0.05; ** *p* < 0.01; *** *p* < 0.001 (all two-tailed).

**Table 2:** Output details from the three hierarchical regression analyses.

Dependent Variable		Criterion Variables	*b*	ΔR^2^	Δ*F*	Δ*p*
Cognitive Risk (RQ5)						
	Step 1:			0.23	5.44	0.001
		Age group	0.53 **			
		Accidents past 12 months	0.03			
		Serious accidents in past 4 years	0.17			
		Perceived control	−0.28 *			
	Step 2:			0.01	0.33	0.801
		Age group	0.30			
		Accidents past 12 months	0.04			
		Serious accidents in past 4 years	0.13			
		Perceived control	−0.31			
		Age by accident past 12 months	−0.08			
		Age by serious accidents	0.23			
		Age by perceived control	0.07			
Intentions (RQ7)						
	Step 1:			0.18	3.89	0.006
		Cognitive risk	0.01			
		Affective risk	0.26 *			
		Self-efficacy	0.12			
		Response efficacy	0.19			
	Step 2:			0.04	0.79	0.539
		Cognitive risk	0.07			
		Affective risk	0.76			
		Self-efficacy	0.15			
		Response efficacy	0.55			
		Cognitive risk by self-efficacy	0.30			
		Cognitive risk by response efficacy	−0.24			
		Affective risk by self-efficacy	−0.32			
		Affective risk by response efficacy	0.12			
Intentions (RQ8)						
	Step 1:			0.32	6.66	>0.001
		Age group	0.37			
		General risk attitudes	−0.15 **			
		Perceived control	−0.02			
		Serious accidents in past 4 years	0.18			
		Accidents in past 12 months	0.02			
	Step 2:			0.06	1.67	0.167
		Age group	0.36			
		General risk attitudes	−0.18 *			
		Perceived control	0.13			
		Serious accidents in past 4 years	0.04			
		Accidents in past 12 months	0.05			
		Age by general risk attitudes	0.09			
		Age by perceived control	−0.14			
		Age by accidents in past 4 years	0.66			
		Age by accidents in past 12 months	−0.17			

* *p *< 0.05, ** *p* < 0.01.
